# Developing pathways for community-led research with big data: a content analysis of stakeholder interviews

**DOI:** 10.1186/s12961-020-00589-7

**Published:** 2020-07-08

**Authors:** Shira Grayson, Megan Doerr, Joon-Ho Yu

**Affiliations:** 1 Sage Bionetworks, 2901 Third Avenue, Seattle, WA 98121 United States of America; 2 Institute for Public Health Genetics, University of Washington, 1959 NE Pacific Street, Seattle, WA 98195 United States of America; 3 Department of Pediatrics, University of Washington, 1959 NE Pacific Street, Seattle, WA 98195 United States of America; 4 Treuman Katz Center for Pediatric Bioethics, Seattle Children’s Hospital and Research Institute, 1900 9th Ave, Seattle, WA 98101 United States of America

**Keywords:** Big Data, community engagement, community-led research, patient-led research, public health, qualitative analysis

## Abstract

**Background:**

Big data (BD) informs nearly every aspect of our lives and, in health research, is the foundation for basic discovery and its tailored translation into healthcare. Yet, as new data resources and citizen/patient-led science movements offer sites of innovation, segments of the population with the lowest health status are least likely to engage in BD research either as intentional data contributors or as ‘citizen/community scientists’. Progress is being made to include a more diverse spectrum of research participants in datasets and to encourage inclusive and collaborative engagement in research through community-based participatory research approaches, citizen/patient-led research pilots and incremental research policy changes*.* However, additional evidence-based policies are needed at the organisational, community and national levels to strengthen capacity-building and widespread adoption of these approaches to ensure that the translation of research is effectively used to improve health and health equity. The aims of this study are to capture uses of BD (‘use cases’) from the perspectives of community leaders and to identify needs and barriers for enabling community-led BD science.

**Methods:**

We conducted a qualitative content analysis of semi-structured key informant interviews with 16 community leaders.

**Results:**

Based on our analysis findings, we developed a BD Engagement Model illustrating the pathways and various forces for and against community engagement in BD research.

**Conclusions:**

The goal of our Model is to promote concrete, transparent dialogue between communities and researchers about barriers and facilitators of authentic community-engaged BD research. Findings from this study will inform the subsequent phases of a multi-phased project with the ultimate aims of organising fundable frameworks and identifying policy options to support BD projects within community settings.

## Background

Big Data (BD; broadly defined as any complex dataset of any size with genomic and diverse phenotypic data, e.g. Fitbit or environmental data) informs nearly all aspects of our lives, including our social, commercial and institutional interactions. In the health sciences and healthcare, BD is the foundation for both basic discovery and tailored translational research — a resource expected to deliver [1–6]. This powerful vision to leverage BD research and BD-derived products to improve the health and wellbeing of society relies on the volume and breadth of the data collected, the representativeness of the datasets, and the processes by which BD research is disseminated and translated into practice [7–9].

Recruitment, engagement and retainment of communities who have been historically underrepresented in biomedical research (UBR) (such as those from racial and ethnic minorities, socio-economically disadvantaged backgrounds or with disabilities), remain areas for improvement across all research disciplines [10, 11]. Studies suggest that the existing barriers to research participation among UBR communities include a lack of awareness about research opportunities [12, 13], a belief that the research is not relevant to themselves or their community [14], limited comprehension of the research purpose or procedures [15], and frustrations due to poor dissemination of research findings back to community members [12]. Some UBR groups are extremely cautious or unwilling to share personal health information with researchers due to mistrust and trauma resulting from recent and historical research malpractice [16, 17]. These barriers to participation can be especially detrimental for ensuring that communities’ priorities are comprehensively addressed, prioritised, and integrated into health research agendas [11, 18, 19].

Progress is being made to include a more diverse spectrum of research participants in datasets [20, 21] and to encourage inclusive and collaborative engagement in research through community-based participatory research approaches [22, 23], citizen/patient-led research pilots [24–27] and incremental policy changes [28, 29]. Specifically, community-based participatory research principles promote “*a collaborative, partnership approach to research that equitably involves community members, organizational representatives, and researchers in all aspects of the research process*” [30]. Citizen/patient-led research pilots aim to make data sources more accessible to interested members of the public in an effort to transfer the ownership, autonomy and decision-making power of the datasets back to those who contribute data and are directly impacted by the research findings [31, 32]. There are also emerging research policies that focus on restructuring grant application and review processes, funding and building capacity for research partnerships, and implementing training and education opportunities for traditional researchers and community members to ensure that communities are engaged in and benefit from health research [30, 33].

These collective efforts offer a promising start to empower groups who have been disenfranchised by the research enterprise. However, additional evidence-based policies are needed at the organisational, community and national levels to strengthen capacity-building and widespread adoption of these approaches to ensure that the translation of research is effectively used to improve the health of communities and address health inequities.

Thus, central to realising the powerful vision of BD-driven tailored health is tangibly building capacity for a movement of community-led BD research science and BD scientists to design BD research and derived products that target health disparities and promote health equity*.* The aim of our study was to detail existing and novel uses of BD (‘use cases’) from the perspectives of community leaders. This study constitutes the first landscape-modelling phase of an ongoing multi-phased project with the ultimate goals of organising fundable frameworks and identifying policy options to support BD projects within community settings.

## Methods

### Study design

We planned semi-structured key informant interviews with the objective to capture ‘use cases’ illustrating possible ways in which communities might interact with BD research and use BD for their benefit. We developed our interview guide iteratively, informed by literature on community-engaged research, citizen/patient-led research and precision medicine. The study protocol was reviewed by the University of Washington Human Subjects Division and determined to qualify for exempt (category 2) status (STUDY00006646).

### Participant eligibility and recruitment

Key informants were identified from our professional networks and invited to participate in a 1-hour interview. Our professional networks include research and community partners engaged in ethics and policy work, community-engaged research and open science collaborations. We intentionally selected participants with whom we had established relationships to maximise trust and openness, increasing the likelihood of collecting authentic data from community leaders. We asked interviewees to provide referrals, leading to snowball sampling and recruitment of additional participants.

All participants were invited by email to join the study and participate in a recorded interview that would be transcribed, de-identified and analysed. Participants were informed that no compensation would be offered in exchange for their participation. Those who responded affirmatively to the email were considered part of the study informant pool and scheduled for an interview.

### Interview procedures

One of two researchers (MD, JHY) led the recorded interviews. Our interview guide prompted participants to describe their community or personal research priorities, how BD might support community priorities, and the types of knowledge, skills and tools needed to achieve these goals. We broadly defined BD as any complex dataset of any size with genomic and diverse phenotypic data (e.g. Fitbit or environmental data). A short demographic survey was sent via email to each participant following their interview.

### Data analysis

Interview transcripts were de-identified using participant identification numbers with other identifiable information redacted. We analysed the transcripts using content analysis, a form of qualitative inquiry that seeks to identify, distil and characterize themes, ideas and topics from various text sources [34]. We derived a coding framework based on our a priori research questions, the interview guide and review of relevant background literature. Emergent themes were also extracted.

The coded dataset was then analysed via an iterative process of decontextualisation and recontextualisation [35]. The primary coder (SG) coded all transcripts and consulted with the two other members of the analysis team weekly to discuss codes, findings and themes over a 3-month period. While our thematic findings reflect the perspectives of community stakeholders interviewed in our study, they are not generalisable to all community stakeholders and we caution such interpretation.

## Results

### Participant interviews

Twenty-one community leaders were invited to participate in a 1-hour interview, 16 of whom responded affirmatively and 2 agreed to participate if needed, but asked to be deprioritised due to their unfamiliarity with the subject; 3 invited leaders did not respond. In total, we conducted 16 key informant interviews between March and May 2019, ranging in length from 44 to 71 minutes (mean: 59 minutes, median: 60.5 minutes). Due to a technical error, 1 interview was not recorded, so detailed notes taken during the interview by investigators MD and SG replaced the transcript in the analysis. Finally, 14 of the 16 key informants completed the demographic surveys, summarized in Table 1.

**Table 1:** Demographics of community leader key informants. Demographic information summarised from the sample of key informants who completed demographic surveys (*n* = 14)

		***n*** **= 14**	
**Characteristics**		**Frequency**	**Percent (%) of study population**^**a**^
**1. Age**	Mean age, years (range; SD)	46 (30–60); 9.5	
	32 and under	1	7%
	33–41	3	21%
	42–54	2	14%
	55 and over	2	14%
**2. Gender identity**	Male	4	29%
	Female	9	64%
	Transgender	1	7%
**3. Ethnicity**	Hispanic or Latino	3	21%
	Not Hispanic or Latino	11	79%
**4. Race**	American Indian/Alaska Native	1	7%
	Asian	3	21%
	Black or African American	1	7%
	Native Hawaiian or other Pacific Islander	1	7%
	White	8	57%
	Other	2	14%
**5. Occupation**	Medical/Genetics/Public Health Researcher, Community Health Advocate, Community Data Organiser, Web Developer, Policy Advisor/Deputy Director, Volunteer/Board Director for Rare Disease Foundation, Consultant, Patient Advocate, Attorney, Physician, Professor		
**6. Highest level of education completed**	Did not complete high school	0	0%
	High school graduate/GED	1	7%
	Some college	0	0%
	College graduate	5	36%
	Post-graduate (e.g. MA, MS, MD, PhD)	8	57%
**7. Statement that best describes knowledge of genetics**	I know nothing about genetics	0	0%
	I remember some information about genetics from school	4	29%
	I am well informed about genetics	10	71%
**8. Community-based organisation?**	Yes	6	43%
	No	5	36%
	Doesn’t apply	3	21%
**9. Description of community or stakeholders**	Community health centres, caregivers and patients of rare disease communities, tribal leaders and community members, non-profit organisations prioritising underserved populations, LGBTQ community, previvors and survivors with hereditary cancers		
**10.Number of employees at organization**	< 10	4	29%
	11 to 50	3	21%
	> 50	4	29%
	Doesn’t apply	3	21%
**11. Organization’s approximate annual operating budget**	< 1 million	4	29%
	1 to 3 million	3	21%
	> 3 million	3	21%
	Doesn’t apply	4	29%
**12. If applicable, populations served and/or represented by organisation**	American Indian/Alaska Native	5	36%
	Asian	5	36%
	Black or African American	3	21%
	Native Hawaiian or other Pacific Islander	5	36%
	White	3	21%
	Hispanic or Latino	4	29%
	Other	3	21%
**13. Ever had a genetic test?**	Yes	9	64%
	No	3	21%
	Don’t know	1	7%
	Pending	1	7%
**14. Ever participated in biomedical research?**	Yes	8	57%
	No	5	36%
	No response	1	7%
**15. Ever participated in genetic research?**	Yes	5	36%
	No	8	57%
	No response	1	7%

^a^Some percentages add to more than 100% due to the ‘select all that apply’ survey option

### Analysis of findings and model description

**Fig. 1:**
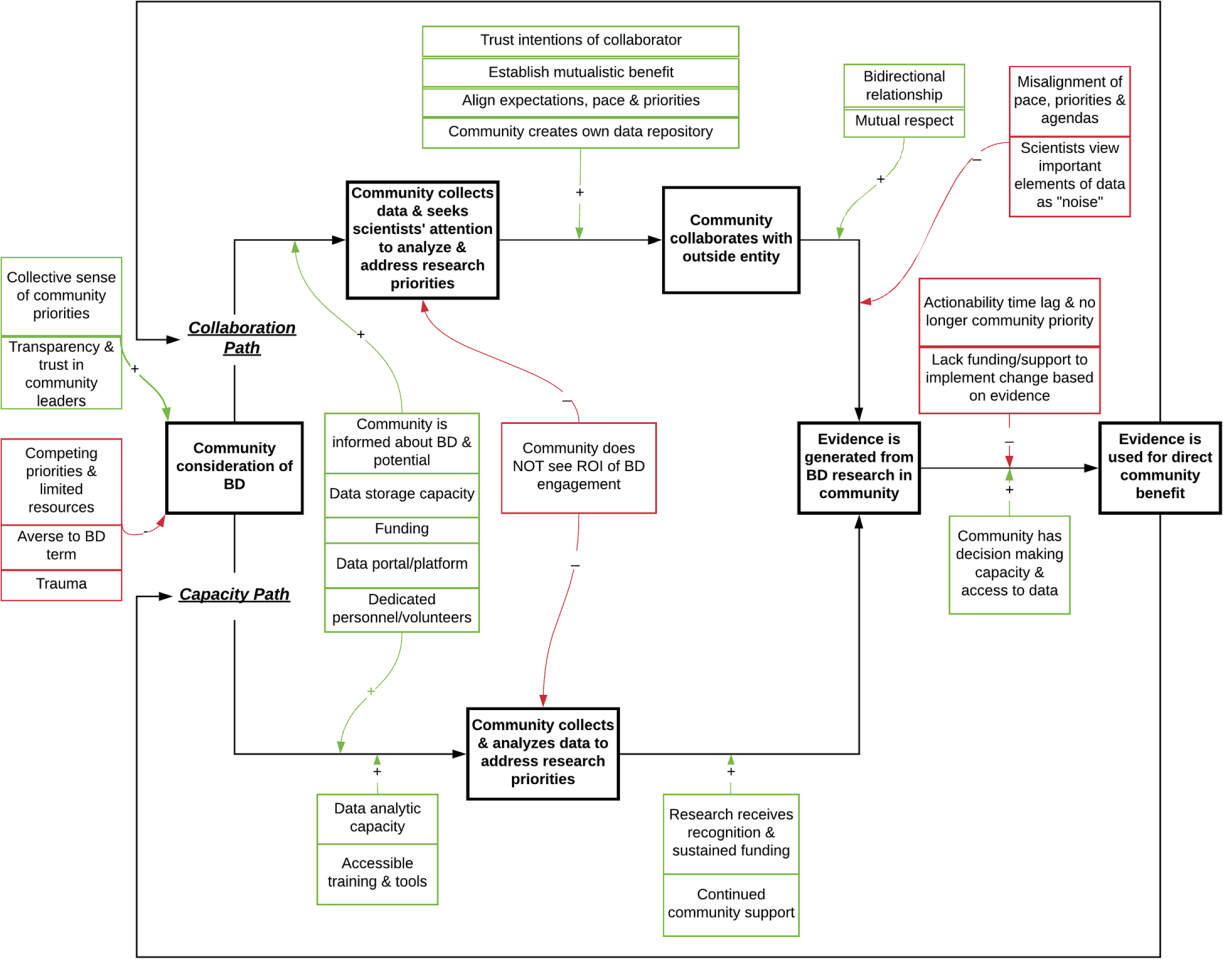
Big Data (BD) Community Engagement Model. The model illustrates the various pathways by which communities engage in BD research, according to analysis findings from 16 key informant interviews. The factors that positively and negatively reinforce actions along a pathway are depicted in green and red, respectively

Key findings from the directed content analysis are summarized in Table 2. Our analysis findings informed the development of the BD Community Engagement Model (Fig. 1). The model describes the processes by which communities move from considering how BD could aid or intersect with community priorities to translating evidence generated from BD research for direct community benefit. The model facilitators (Fig. 1, in green) represent factors that positively reinforce actions along a pathway and the model barriers (Fig. 1, in red) represent factors that negatively reinforce actions along a pathway, based on our analysis findings.

**Table 2:** Summary of responses by a priori content codes. Key findings from the directed content analysis of key informant interviews (*n* = 16) summarized by content codes: Use cases, Desires and visions, Tools and supports, Barriers, Facilitators, and Attitudes

**Content code**	**Summary of responses**
**Use cases**	• Improve screening, treatment and prevention options • Nuanced risk prediction and decision-making tools • Investigate broad range of health determinants • Provide community agency via data access
**Desires and visions**	• Legitimise career paths for citizen scientists and patient advocates • Improve science and health literacy • Authentic collaborations • Accessible and affordable genetic tests
**Tools and supports**	• Mentorship and partnerships • Reliable and sustainable funding sources • Data tools and training • Data sharing platforms
**Barriers**	• Disconnect between healthcare, research and community needs • Data capacity challenges • Competing community priorities • Unfamiliarity with Big Data and research • Fear, trauma and mistrust associated with research experiences
**Facilitators**	• Collaboration frameworks and shared resources • Interoperability and centrality of data • Trust in leaders and political will
**Attitudes**	• Data are valuable and personal • Big Data currently lacks bidirectionality • Superficial community engagement • Data interpretation requires cultural context

### Community consideration of BD

In the first step of our model, a community considers how BD might aid or intersect with its priorities and needs. This step represents a distinct ‘snapshot’ in time for some informants, such as those from rare disease communities, who actively seek BD as a solution to uncover as much information as possible after receiving a rare diagnosis. Other informants shared how BD passively diffused into their community over the span of several months or years in the absence of an initiating event or action. One informant described how this process was eventually formalized to become a “*gathering every year to talk to the community,* [to hear] *their priorities, and put out all the major data*” [P10].

One key factor for facilitating communities’ capacity to invest in new programmes, ideas or research endeavours, such as BD, is establishing a collective sense of community priorities. Based on an informant’s experience, “… *until there is a sense of congealing and collective sense of vision, and goals, and priorities, then* [the community] *is this kind of multi-armed octopus that’s just kind of going in so many different research directions*” [P3].

Most leaders in our informant pool expressed interest in building capacity for BD research within their communities but faced competing community priorities and limited resources that threatened their ability to move past this initial stage in the model. One informant clearly explained this dilemma,

“*From a very idealistic perspective, I think having that in-house capacity for community-based organizations to be able to generate, contribute, analyze, and utilize their own data to contribute to the broader research field would be really, really powerful. But I also from a pragmatic perspective recognize that organizations are just struggling to stay afloat. And so, finding resources to support staffing to actually just provide the services is a very real challenge for our communities*” [P3].

Similar sentiments were echoed by an informant who revealed how the community may be intrigued by the potential of BD research in theory, but “… *what’s so hard in UBR populations* [is] *they’re not only worried about health. They’re worried about stuff that a lot of us don’t worry about* [...] *They’re worried about their day to day safety and neighborhoods are not safe. And you come to talk to me about Big Data? It’s like take a seat. Take a number*” [P11].

Another important barrier that surfaced during our interviews was that some communities viewed the term ‘Big Data’ as an elitist and exclusionary buzz word. An informant who expressed an aversion toward this term interjected mid-way through their interview, “*Whenever you say big data, like I just kind of zone out. Big data feels like not relevant to me but now I see that big data is relevant to us … I never think of* [Facebook, Google, or Amazon data] *as big data. But that’s exactly what we would like to be doing*” [P15].

Several of our informants proposed increasing community familiarity with and comprehension of BD and its implications as a key strategy for facilitating community interest in BD. Other community leaders cautioned that these efforts would only aid communities who already have access to the technology required for generating BD, consequently widening the digital divide and excluding certain communities from collecting and analysing their own BD. One tribal leader offered a counter argument to this perspective explaining, “*for now, maybe* [a lack of access to technology] *is not such a bad thing because too often what we see is a rush in the technology and a huge lag in the ethics and the policy discussion afterwards. And at least even there are barriers to collecting information in tribal communities, at least it’s not outpacing our discussion.*” [P16].

The emotional trauma that communities experienced due to a history of research malpractice and/or a lack of transparency serves as another significant barrier for community consideration of BD endeavours. This trauma, “… *makes* [our group] *not only reluctant and distrustful of participating in big data, but it also makes them very vulnerable to big data because it gets collected anyway and often they don’t know it’s happening*” [P6]. Informants described how challenging it is to regain trust, “*Some of these communities were never informed … They were basically, you know, thrown into a study … Their individual concerns were never part of the equation. So now, we’re saying, ‘Oh, you matter’. And they’re saying, ‘You never said that before’. And so, you’re asking them to trust again, so the how and the why are very, very formidable challenges*” [P11]. Another form of emotional trauma shared in our interviews was this notion of ‘academic exclusion’, wherein community members were “*… told in school they were less than*” [P9].

Some informants highlighted that UBR communities may also feel a certain level of vulnerability when engaging with BD research because of the perceived risks of being targeted and profiled based on this information,

“*There’s been increasing concern about data tracking, about social media, about the ways in which so-called gang databases are put together using big data. So, there’s like increasing mistrust.* […] *It feels really vulnerable. And for young people of color who are at risk of being targeted and profiled, it feels really dangerous in some ways. And when you think about data, and you think about translation, and you think about use and application, you know, culture matters and cultural competency matters*” [P9].

What is clear from our analysis is that communities may have faced a range of barriers and may or may not have experienced any facilitators when considering BD research. We repeatedly heard from our informants that the interviews felt like a safe environment to unload pent up trauma, vulnerability and frustrations from previous research involvement. For example, one informant admitted late in the interview, “*I tend to think that I’m not gonna be very useful because I’m not really a professional, but then I was like …* [this interview is] *kind of like therapy for me. So, thank you for listening*” [P15]. Further, some communities may have progressed past this stage of considering BD engagement at one point in time but may have returned to this stage due to myriad experiences, including failed collaborations, re-traumatisation, misuse of data, reprioritisation of community needs or broken trust with community leaders.

### To engage or not to engage, that is the question

Communities who conceptualise and wish to act on opportunities to use BD for community benefit, tend to follow the ‘Collaboration Path’ or the ‘Capacity Path’ in the model (Fig. 1)*.* The ‘Collaboration Path’ represents the experience of a community who collects its own data and seeks the attention of qualified scientists to analyse its data and address community research priorities. The ‘Capacity Path’ represents a community whose members collect and analyse their own data and independently address community research questions.

The ‘Collaboration’ and ‘Capacity’ paths are both facilitated by community knowledge of BD and its potential applications, data storage capacity, funding to sustain the community organisation, and dedicated personnel and volunteers committed to overseeing a BD project. If a community does not see a potential return on investment from engaging in BD research, this becomes a barrier affecting both pathways. A recommended tactic to overcome this barrier is to offer “… *specific examples for the community* [so] *that they can easily understand what is the value to be part of a research study. It’s not only to say that you will contribute with your information to create the data to make available for the researchers* […] *then what happens? Something that will be important* […] *is to provide results at the high level of the project to the participant*” [P4].

The primary difference between the two pathways is the decision made by communities on the ‘Collaboration Path’ to engage an outside entity (e.g. an organisation, health centre, data scientist, foundation) to form a research collaboration. This step created one of the greatest bottlenecks within this model, requiring significant effort on the part of the community. In fact, many reported turning to the ‘Capacity Path’ after a failed attempt along the ‘Collaboration Path’. One informant admitted, “*I really don’t have a lot of interaction with* [professional scientists]. *And at this point, like I don’t even trust them. I’ve been working at this for long enough that if they just turned around and suddenly wanted to work with me, I don’t really wanna work with them at this point, but that’s been a huge bottleneck for us*” [P15].

Community leaders repeatedly stressed the need for help from professional scientists. “*You know, what it takes me 10 hours to do, somebody who had a PhD could probably do in a half hour with their eyes shut*” [P8]. At the same time, informants reported being exploited by professional scientists. “*It’s just we keep beating our heads against this wall and making new changes with bubble gum and tape and no resources, no PhDs, and then everybody writes the papers about us. Everybody goes off and builds the platforms based on our ideas. And nobody ever actually partners with us*” [P1].

One community leader explained that the onus is primarily on the community to be informed and do their research about the rules of collaborations in order to appear credible and attractive to a collaborator, “*As a patient group or as a community, you’ve got to make yourself approachable, attractive, and a credible partner who’s willing to play the give and take game of every relationship. You have to know when you need to compromise and when you cannot compromise … You’ve got to be really well informed … it all begins with mutual respect and trust*” [P11].

Yet, many informants identified the current research infrastructure as a key impediment to meaningful bidirectional collaboration. “*Doing community engagement is a totally different skillset. And it’s tough and that’s not how* [traditional researchers] *get promoted. They get promoted in the academic space based on publications. And so, the incentives are not lined up to say, ‘Hey, let’s take a risk. Let’s try to do this differently and work* [directly with communities]*’*” [P12].

Another informant explained, “*There’s been this kind of idea attached to funding and I’m starting to actually see it like written into grants for researchers that you involve patients, I’m seeing it done in the most superficial ways* […] *and it’s very frustrating to watch because it’s like you’re being told you have something, but you don’t actually have it*” [P15].

Even communities with established research collaborations expressed challenges related to aligning perspectives on the important elements of the datasets and the analytic approaches employed to address the questions most relevant to the patient population. One leader shared how, “*traditional researchers are just like way back like 3 years ago of where we are in terms of understanding how rich the* [data are] *… and they’re kind of just like getting accustomed to how rich the data is and they still haven’t really understood all the potential of it*” [P2]. Another leader elaborated on how, “*It’s a huge amount of work to onboard a data scientist to really understand the* [disease] *context and the data context and also data scientists who are traditionally trained have traditional methods and want things to fit in nice boxes and be clean. What we learned is … we have new problems that people haven’t been able to analyze before. So, this is going to involve new methods of analyses*” [P1].

A common thread expressed by community leaders on the ‘Collaboration Path’ was an interest in improving what we have termed the ‘authenticity’ of research collaborations, referring to research relationships formed and sustained based on bidirectional benefit shared between researchers and the community, without coercion or contradicting community values. One informant described their experience advising a fellow community leader against what they viewed as an inauthentic collaboration, “*I pointed out the clause about patient involvement and I was like this grant is requiring patient involvement. The researcher … had already designed and conceived of the entire thing. They haven’t actually like worked with her to develop the idea. They had the idea. They had fleshed it out and then I think they felt like they were gifting her with the opportunity to partner on it.*” [P15].

Communities on the ‘Capacity Path’ translate their collected data to address their research priorities if their work is recognised as legitimate by the scientific establishment, sustained funding is available, and the community remains engaged in the work. One informant described their difficult experience working to translate their research and receive recognition for their work in the absence of outside collaboration, “*All of us in the … movement know each other. And we’ve all done this work. And we clawed our way to, like, find some sense of a path for ourselves that is never ever valued. I mean, we’ve created all this value in the healthcare system through patient engagement. So, for us to wake up and recognize how little that is actually respected or how little rights we really have after all the work we’ve done, it’s hard for everybody, I’ll tell you. And so, in my mind, we do need to create a path to legitimacy*” [P1].

The overwhelming feedback we heard from informants supported our observation that the decision between the ‘Collaboration Path’ and the ‘Capacity Path’ is not necessarily calculated. Most communities are initially interested in the ‘Collaboration Path’, yet only a select few successfully establish the type of authentic collaborations necessary for continuation along this pathway.

### From evidence generation to direct community benefit

A minority of key informants expressed that they had ever reached this final stage of returned community benefit from BD research. This final step is facilitated only if a community can access their data. As one informant explains, “*Just like the old adage that possession is 9/10 of the law, possession of the data gives you a lot of leverage …*. *If you don’t have the data to show … then you can’t go to Congress and lobby on your own behalf*”. This informant reflected on how this directly impacted their community, “*There were 5 tribes that went to court against the army corps of engineers. The reason why they lost is they couldn’t find the data. There’s data out there, but it wasn’t accessible to the tribes even though it was collected on reservation land and it was presumably for reservation benefit*” [P6].

Informants frequently referenced the importance of having decision-making capacity and an understanding of culture and context in order to translate data into community benefit. Because, “*possessing the data isn’t enough. Just because you have that data, if you don’t have a sense of self-efficacy or self-determination to be able to advocate it for yourself,* […] *then having that data is of no use*” [P3].

A key barrier identified at this stage is when communities lack the funding or support to plan, install and implement change based on the evidence generated. One frequent example expressed by informants was the dearth of resources and capacity to plan for, troubleshoot, and deal with poor data interoperability. A community leader pointed out how generating data does not ensure the data are accessible in a useful format for deriving community benefit, “*It’s the interoperability of the data and the real time and retrospective access to it. Those were the key components that unlock everything else. And I feel like those are the ingredients for the recipe of the success for any community, even one with less resources and even ones that are considered to be less data driven. We’ll get a ton of insight if we can free the data to the right people who can say, ‘Aha, here’s the problem and here’s the solution based on what the data is telling us’*” [P2].

Our findings highlight that when communities experience a return on investment from engaging in BD research (depicted in this final stage of the model), this often strengthens communities’ sense of autonomy, self-governing capacity and empowerment, which in turn may facilitate reengagement in this BD pathway. One informant summed up this phenomenon, “*The only thing we can control is our own choices. And to me, that’s what autonomy is. And our individual choices and our collective choices as a group are the one thing I’m trying to protect right now to be honest... If we don’t, like, have autonomy over our data, over our community, and all of these* [players] *in big data start making decisions about us without us*” [P1].

## Discussion

This semi-structured content analysis of community stakeholder interviews sought to detail existing and novel intersection points for communities with BD research (‘use cases’). Based on our review of relevant literature and our work in community-engaged research, ethics and policy, and open science collaborations, we anticipated to hear from stakeholders about BD barriers, including distrust and privacy concerns with personal identifiable information [36], fears of data misinterpretation due to missing information [37], barriers to data access and sharing [38], lack of data science training and resources for data analysis and interpretation [39], and trauma from a lack of transparency and a history of research malpractice [16]. Our findings support the existence of these barriers and also illuminate when and how these barriers are fortified.

Additionally, we saw strong evidence of the consequences of the ‘digital divide’ defined as inequalities that arise due to an uneven distribution of access to information or technology and, in the context of BD, inequalities between data donors and those who analyse and interpret the data [40–42]. This concept is especially relevant given that BD collection and analysis often include electronic health record data, clinical registries, lab tests, insurance claims and genomic data [43]. This inequality is particularly salient, as the vast majority of the communities in our study did not have the necessary infrastructure or the advanced analytic training to fully understand how their community’s data would be used or analysed. This appeared to perpetuate mistrust of researchers, a disinterest in BD engagement, and a fear that a community’s data might be used inappropriately. Most informants discussed how these gaps in data/scientific literacy posed significant barriers for generating interest in BD research.

One finding we did not anticipate from our interviews was that the term ‘Big Data’ was viewed as elitist, inaccessible and irrelevant by communities, further deterring engagement in BD research and reinforcing this digital divide. Another novel finding was a ‘positive’ interpretation of the digital divide — that a community’s lack of access to technology might mean there is little BD for outsiders to mine. This form of ‘invisibility’ renders communities less vulnerable and can enable them to build their internal knowledge and capacity prior to engaging with BD research [44]. Conversely, although avoiding the potential harms of BD may be a reasonable protectionist strategy in the early-phases of BD research, an absence from BD could indefinitely defer potential benefits from BD insights in an age of precision medicine.

A fundamental challenge for communities was establishing and sustaining authentic research collaborations, which we defined as research relationships formed and sustained based on bidirectional benefit shared between researchers and the community, without coercion or contradiction of community values. Research findings support that community involvement at all stages in the research process increases the quality, relevance and efficacy of research translation to improve individual and population health [20, 21]. However, determining how best to engage public participation in research remains an ongoing challenge for researchers who are not traditionally trained to identify, recruit and convene stakeholders to facilitate this type of engagement [45]. Our informants repeatedly described how naive and self-serving attempts by researchers to facilitate engagement efforts discouraged communities from reengaging in future research collaborations. Unfortunately, we heard that current research infrastructure, policies and incentives are not aligned to support community engagement work and this is often a root cause of failed community engagement efforts [46, 47].

Some research sponsors aim to address this issue by creating more structured and accountable methods for obtaining input from stakeholders on research design, conduct and dissemination of findings [15, 21, 47]. However, our community stakeholders described that these existing support systems, meant to encourage community/patient engagement in research (e.g. via grant requirements), often involve superficial community engagement efforts that are sometimes initiated after the study design has already been finalised by the researchers. Several community leaders found themselves entangled in this type of inauthentic or ‘parasitic’ research relationship where researchers who directly benefited from their community’s research participation and shared data deprioritised or intentionally ignored the community’s needs. The number of informants who described the interviews as ‘therapy sessions’ demonstrated just how traumatising these parasitic research relationships can be for communities. Ironically, this ‘research parasite’ trope has more famously been used by researchers in the context of open data-sharing platforms to describe citizen/community scientists who use a research group’s data for their own ends [48]. However, in our interviews, the researchers were painted as ‘research parasites’, which suggests a need to collectively redefine the scope and parameters of community relationships with researchers who typically hold the locus of power and control in research. This finding also highlights a possible avenue for exploring ‘soft-skills’ and/or training for traditional researchers to equip them for working more effectively in community settings.

As a synthesis of our informants’ experiences, the BD Community Engagement Model illustrates the pathways and various forces for and against community engagement in BD research. Our research findings support that BD engagement is an iterative process that occurs in stages along different trajectories as opposed to a single destination. Rarely did informants express that they had reached the final stage of returned community benefit from BD research, which highlights the fallacy that, when data are collected, they intrinsically provide value. Our model was intentionally designed to reflect the mobility of communities forward as well as in retreat along BD engagement pathways, reflecting both positive and negative community experiences with BD to broadly contextualise the arc of the engagement process. Our intention is for the model to serve as tool to promote concrete, transparent dialogue between communities, researchers, policy-makers, heath practitioners and society at large about the barriers and facilitators of authentic community-engaged BD research.

Although not captured during our interviews, we recognise that there is also the potential for communities to experience a ‘middle-way’/‘alternative’ pathway that represents a blend of the ‘Collaboration’ and ‘Capacity’ paths depicted in our model. Based on our analysis, we envision that this pathway might involve communities spearheading and owning their own research projects supported by (but not necessarily led by) professional researchers and open resources. We also acknowledge that the metrics for ‘successful engagement’ may differ greatly by stakeholder (i.e. community leader versus patient advocate versus research funder). Thus, in addition to discussing the identified barriers and facilitators for BD research with a range of stakeholders, we must also remain open to exploring alternative ‘middle-way’ pathways for enabling communities to derive the most benefit from and autonomy within BD research.

## Conclusion

Diverse informants across the fields of citizen science, patient-led research and community-based organisations shared comparable stories of frustration and challenge in engaging in research across vastly different health issues and constructs of community. In order for communities to derive benefit from and find autonomy within BD research, their voices, values and priorities must be represented and recognised throughout the entire research process, from data collection to research translation. We hope that the model amplifies key challenges relevant to community engagement and autonomy within BD research. In the next phases of this project, we will incorporate our model findings and additional input from stakeholders to identify concrete policy options and tangible tools that support more equitable research participation and health outcomes.

## Data Availability

The authors welcome correspondence regarding the dataset they gathered for this study and their analysis of the dataset. They will consult with the IRB of record regarding any requests to review these materials as the participants of this study were not consented for broad data sharing.

## Abbreviations

BD: Big Data
UBR: underrepresented in biomedical research
